# Severely Circumferentially Calcified Neointima as a New Cause of Undilatable In-Stent Restenosis

**DOI:** 10.1155/2018/5764897

**Published:** 2018-05-29

**Authors:** Manabu Kashiwagi, Takashi Tanimoto, Hironori Kitabata

**Affiliations:** Department of Cardiology, Shingu Municipal Medical Center, Shingu, Japan

## Abstract

A 74-year old man presented recurrent angina pectoris due to in-stent restenosis (ISR) with severely calcified neointima. In-stent neoatherosclerosis (NA) is associated with late stent failure, and NA with calcified neointima occurs in some cases. Because the presence of neointimal calcification could lead to underexpansion of newly implanted stent for ISR, a scoring balloon was selected for predilatation to obtain maximum extrusion of the neointimal plaque and subsequently, an everolimus-eluting stent was implanted. However, moderate stenosis remained on coronary angiography, and optical coherence tomography (OCT) revealed underexpansion of the newly implanted stent because an attempt at balloon dilatation of neointimal calcification failed. Although OCT can clearly discriminate stent struts from neointimal calcification, we did not perform OCT assessment between scoring balloon and stenting. It is highly recommended to confirm whether the lesion is adequately treated by balloon angioplasty before stenting in cases with calcified ISR.

## 1. Introduction

Second-generation everolimus-eluting stent for in-stent restenosis (ISR) of drug-eluting stents (DES) is considered as an effective approach although some patients still have recurrences [1]. Currently, paclitaxel-coated balloon (PCB) has also been proposed as an alternative therapy for patients with ISR and could prevent target lesion revascularization better than conventional balloon angioplasty [2]. Although neointimal calcification (NC) is not frequent, the presence of NC could lead to underexpansion of newly implanted stent for ISR [3]. We therefore presented a patient with unfavorable acute result after intervention for DES-ISR with NC.

## 2. Case Presentation

**Figure 1:**
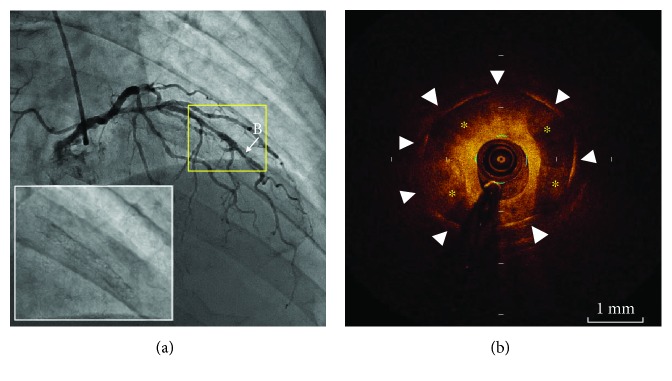
First time coronary angiography and corresponding OCT image. (a) Angiography demonstrated tandem stenotic lesions within the previously implanted stent. (b) OCT revealed severely calcified neointima (asterisks) within well-expanded stent struts (arrowheads) at the distal stenotic site. OCT: optical coherence tomography.

**Figure 2:**
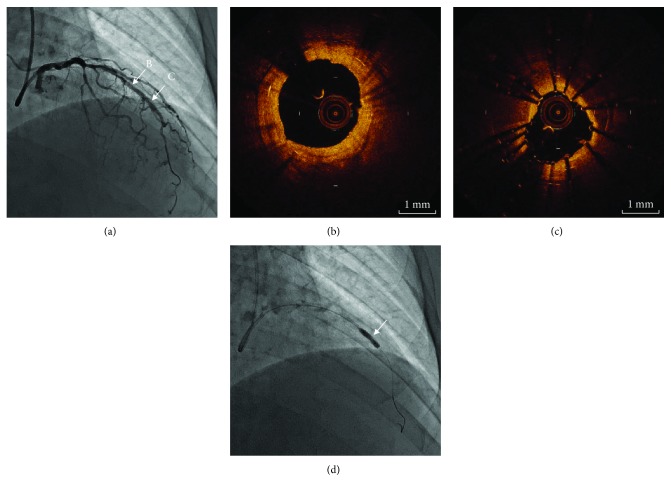
Coronary angiography and corresponding OCT image after PCB and DES implantation. (a) Angiography showed residual stenosis at the newly stented site. (b) OCT image of the lesion treated with PCB. (c) Ununiformed dilatation of newly implanted DES and dilation failure of calcified neointima. (d) Postdilatation with noncompliant balloon. White arrow: balloon indentation. OCT: optical coherence tomography, PCB: paclitaxel-coated balloon, DES: drug-eluting stent.

A 74-year-old man was admitted to our hospital because of effort angina pectoris, and a 2.75 × 24 mm paclitaxel-eluting stent (PES) was implanted at the midportion of left anterior descending artery. After the initial procedure with stent implantation, he underwent repeated angioplasty with cutting balloon due to ISR 4 and 8 months later. These two repeat angioplasties were effective and improved his symptoms. After 5 years, however, he suffered from recurrent chest pain. His coronary risk factors were hypertension and diabetes mellitus (DM). Although aldosterone receptor antagonist was prescribed for hypertension, DM was followed with no medication. His hemoglobin A1c level was 6.3%, and serum levels of low-density lipoprotein was 122 mg/dl. Coronary angiography determined the tandem stenotic lesions within the previously implanted PES as shown in Figure 1(a). Optical coherence tomography (OCT) revealed excessive neointimal hyperplasia within well-expanded stent struts at the distal site (Figure 1(b)). The neointima appeared as a signal-poor and heterogeneous region with s sharply delineated border on OCT image, suggesting a calcified lesion. Therefore, a 2.5 × 13 mm scoring balloon (Lacross NSE; Goodman, Nagoya, Japan) was selected for predilatation to obtain maximum extrusion of the neointimal plaque [4]. Subsequently, a 3.0 × 20 mm PCB (SeQuent Please; B. Braun Melsungen AG, Vascular System, Berlin, Germany) was utilized for the proximal lesion, and a 2.25 × 18 mm everolimus-eluting stent (Xience Xpedition; Abbott Vascular, Santa Clara, California) was implanted at the distal lesion. Although the proximal lesion was well treated with PCB, the coronary angiography still demonstrated moderate stenosis at the distal lesion (Figures 2(a) and 2(b)). OCT revealed underexpansion of the newly implanted stent because an attempt at balloon dilatation of neointimal calcification failed (Figure 2(c)). A 2.5 mm noncompliant balloon was therefore applied for postdilatation, but it was not succeeded. Furthermore, a bigger size noncompliant balloon of 2.75 mm at high inflation pressure (24 atm) did not work well (Figure 2(d)). Even though underexpansion of the newly implanted stent and moderate stenosis were still present (minimum lumen area 2.39 mm^2^), we had to terminate the procedure to avoid the risk of perforation.

## 3. Discussion

Neoatherosclerosis (NA) is encountered at the late phase of implanted coronary stent because coronary stent evokes an inflammatory reaction [5]. Pathological study revealed that the incidence of NA is 31% in DES and 16% in bare-metal stent (BMS) [6, 7]. Moreover, two OCT studies reported that NA with calcified neointima occurred in about 10% cases [8]. Although calcified neointima is negligible, there have been few reports to date focusing on in-stent restenosis with calcified neointima [9]. Severely calcified neointima is occasionally undilatable by balloon angioplasty only and may lead to resistant ISR, requiring rotational atherectomy [10, 11]. In our case, preintervention OCT images indicated the heavily calcified tissue within the stent at the distal lesion. As a result, we should have confirmed whether the lesion is adequately treated by balloon angioplasty before stenting because residual underexpansion of implanted stent could contribute to recurrent ISR and stent thrombosis.

## 4. Conclusion

The inadequate modification of NC led to underexpansion of newly implanted stent for ISR. It is highly recommended to confirm whether the lesion with NC is adequately treated by balloon angioplasty before stenting.

## References

[1] Alfonso F., Pérez-Vizcayno M. J., Cárdenas A. (2015). A prospective randomized trial of drug-eluting balloons versus everolimus-eluting stents in patients with in-stent restenosis of drug-eluting stents: the RIBS IV randomized clinical trial. *Journal of the American College of Cardiology*.

[2] Lee J. M., Park J., Kang J. (2015). Comparison among drug-eluting balloon, drug-eluting stent, and plain balloon angioplasty for the treatment of in-stent restenosis: a network meta-analysis of 11 randomized, controlled trials. *JACC: Cardiovascular Interventions*.

[3] Mehanna E., Attizzani G. F., Nakamura D. (2016). Impact of neointimal calcifications on acute stent performance during the treatment of in-stent restenosis. *Arquivos Brasileiros de Cardiologia*.

[4] Ashida K., Hayase T., Shinmura T. (2013). Efficacy of lacrosse NSE using the “leopard-crawl” technique on severely calcified lesions. *The Journal of Invasive Cardiology*.

[5] Inoue K., Abe K., Ando K. (2004). Pathological analyses of long-term intracoronary Palmaz–Schatz stenting. *Cardiovascular Pathology*.

[6] Nakazawa G., Otsuka F., Nakano M. (2011). The pathology of neoatherosclerosis in human coronary implants: bare-metal and drug-eluting stents. *Journal of the American College of Cardiology*.

[7] Taniwaki M., Windecker S., Zaugg S. (2015). The association between in-stent neoatherosclerosis and native coronary artery disease progression: a long-term angiographic and optical coherence tomography cohort study. *European Heart Journal*.

[8] Takano M., Yamamoto M., Inami S. (2009). Appearance of lipid-laden intima and neovascularization after implantation of bare-metal stents: extended late-phase observation by intracoronary optical coherence tomography. *Journal of the American College of Cardiology*.

[9] Yoshida K., Sadamatsu K. (2012). A severely calcified neointima 9 years after bare metal stent implantation. *Cardiovascular Revascularization Medicine*.

[10] Alfonso F., Sandoval J., Nolte C. (2012). Calcified in-stent restenosis: a rare cause of dilation failure requiring rotational atherectomy. *Circulation: Cardiovascular Interventions*.

[11] Bastante T., Rivero F., Cuesta J., Alfonso F. (2015). Calcified neoatherosclerosis causing “undilatable” in-stent restenosis: insights of optical coherence tomography and role of rotational atherectomy. *JACC: Cardiovascular Interventions*.

